# Perspectives on Viral RNA Genomes and the RNA Folding Problem

**DOI:** 10.3390/v12101126

**Published:** 2020-10-05

**Authors:** Susan J. Schroeder

**Affiliations:** 1 Department of Chemistry & Biochemistry, University of Oklahoma, Norman, OK 73019, USA; susan.schroeder@ou.edu; Tel.: +1-405-325-3092; 2 Department of Microbiology & Plant Biology, University of Oklahoma, Norman, OK 73019, USA

**Keywords:** encapsidated viral RNA, viral RNA folding, structural virology

## Abstract

Viral RNA genomes change shape as virus particles disassemble, form replication complexes, attach to ribosomes for translation, evade host defense mechanisms, and assemble new virus particles. These structurally dynamic RNA shapeshifters present a challenging RNA folding problem, because the RNA sequence adopts multiple structures and may sometimes contain regions of partial disorder. Recent advances in high resolution asymmetric cryoelectron microscopy and chemical probing provide new ways to probe the degree of structure and disorder, and have identified more than one conformation in dynamic equilibrium in viral RNA. Chemical probing and the Detection of RNA Folding Ensembles using Expectation Maximization (DREEM) algorithm has been applied to studies of the dynamic equilibrium conformations in HIV RNA in vitro, in virio, and in vivo. This new type of data provides insight into important questions about virus assembly mechanisms and the fundamental physical forces driving virus particle assembly.

## 1. Introduction

Michael Rossman’s lab’s diffraction images of Southern Bean Mosaic Virus particles revealed beautiful icosahedral symmetry for the protein shell [1], but the structure of the RNA genome inside remained a mystery. The assumption often was that the viral RNA genome was completely disordered. The first crystal structures to reveal well-ordered RNA genomes showed density for RNA helices on the symmetry axes of the icosahedral particle, but the symmetry averaging used to solve the crystal structure obscured the details of the RNA sequences in the helices [2,3]. Since the first observations of encapsidated RNA genomes, there have been many approaches to determining and modelling the structures of viral RNA genomes. This review will focus on two new techniques, asymmetric reconstructions from high resolution cryoelectron microscopy [4,5] and DREEM (Detection of RNA folding Ensembles using Expectation Maximization) [6], that probe the structures of encapsidated RNA. These techniques provide positive evidence that encapsidated RNA forms more than one structure inside a virus particle. This is important information for understanding virus assembly mechanisms and the fundamental physical forces that stabilize virus particle structures.

**Figure 1:**
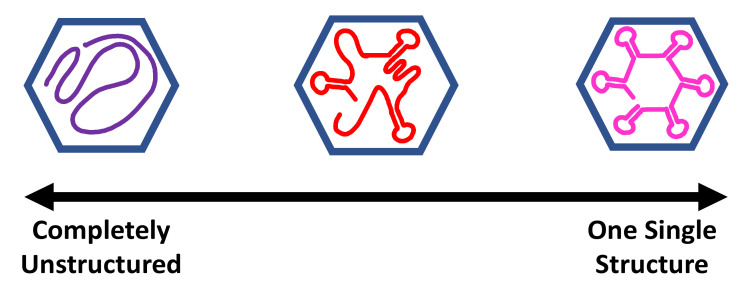
Spectrum of models for encapsidated viral RNA genomes. The blue hexagon represents the symmetric coat protein shell. The purple line represents a completely disordered and unstructured RNA. The red line represents a partially ordered ensemble of RNA structures with some hairpins and some disordered regions. The pink line represents an RNA in a single minimum free-energy structure with well-defined RNA helices.

The current models for encapsidated viral RNA range from completely disordered to a single, well-defined minimum energy structure. Figure 1 shows this spectrum of models for viral RNA genome structures. There are many possible ways to have regions of partial disorder, and partially ordered structures or dynamic structures in the middle region of this spectrum. DREEM and asymmetric reconstructions from cryoelectron microscopy support the idea that the RNA genome has some well-defined functional structures but is also partially disordered, and this is best described as a structural ensemble somewhere in between disordered and a single structure. These techniques have the potential to resolve conflicting models for encapsidated viral RNA genomes.

The different models for encapsidated viral RNA genomes in several viruses have different assumptions about the degree of order underlying the approach to structure elucidation. For example, three models have been proposed for the RNA genome of Human Immunodeficiency Virus: a model focused on a structured 5′UTR [7], an ensemble of RNA structures [6,8], and a single minimum free energy structure model [9]. Similarly, models for Dengue virus genomes have been proposed as a model with regions of well-ordered and dynamic structures [10], or as a single minimum free energy structure [11]. Different models for Satellite Tobacco Mosaic Virus RNA have proposed an ensemble of structures [12] or a single minimum energy model [13,14]. Several models for MS2 bacteriophage RNA have proposed an ensemble of structures [15], models with regions of order and regions of disorder [4,5], or single minimum free energy structures [16].

The models for encapsidated viral RNA may also incorporate hypotheses about the virus assembly mechanism. Many models include a series of hairpin loops which can serve as packaging signals that bind proteins and direct virus particle assembly, for example MS2 [16], STMV [17], and STNV [18]. The packaging signals form functional conserved structures and the sequences connecting the packaging signals may be less well-ordered, which presents another way that viral RNA genomes can be partially ordered and land in the middle of the spectrum from unstructured to structured. On the other hand, models of electrostatic collapse for virus assembly [19,20,21] promote the idea that the encapsidated RNA is largely disordered initially and lies closer to the completely unstructured end of the spectrum.

All of these models use a variety of information from crystallography, cryoelectron microscopy, phylogenetic sequence alignment, RNA nucleotide chemical probing experiments, and thermodynamic-based RNA prediction tools. RNA crosslinking and immunoprecipitation strategies have also been employed to identify encapsidated viral RNA-coat protein interactions [16,22,23]. How the experimental data and free-energy minimization structure predictions are combined depends on the modelling approach, models for virus assembly, and assumptions about the degree of order and disorder in the viral RNA genome. Fortunately, new techniques in asymmetric reconstructions from electron microscopy and DREEM can provide positive evidence to disprove either extreme of the spectrum in Figure 1, better define the degree and type of partial order in encapsidated viral RNA ensembles, and potentially resolve conflicting models of encapsidated viral genomic RNA.

## 2. High Resolution Asymmetric Reconstructions Provide New Insights into Viral RNA Genome Structures

The recent advances in cryoelectron microscopy techniques have opened a new window to look at the ways that viral RNA genomes can be partially ordered and/or dynamically structured inside a viral capsid. The improvements in direct electron detectors, image contrast, and single particle tracking have made high resolution asymmetric reconstructions of virus particles possible [24]. Importantly, the computational techniques to classify images have made possible the study of macromolecular assemblies that exist in more than one conformation or change conformations. In the case of icosahedral virus particles, the outside surface of the virus particle is symmetric and prevents the experimental purifying of different internal conformations of the viral genome. Two examples of high-resolution asymmetric reconstructions of MS2 bacteriophage [4] and Brome Mosaic Virus (BMV) RNA genomes [5] highlight two different models for viral genome structure and virus assembly mechanisms.

Most recently, the high-resolution asymmetric reconstruction of BMV shows a partially ordered ensemble of conformations for the viral RNA genome [5]. BMV has 4 distinct RNAs that compose its genome. BMV packages RNA 1 and RNA 2 in separate T = 3 virus particles while packaging RNA 3 and RNA 4 together in one virus particle. This study generated virus particles from a plasmid expression system in plant leaves so that the virus particles only contain RNA 3 and RNA 4. The asymmetric reconstruction at 3.9 Å resolution shows that the N-terminus of the hexameric conformation of the capsid protein interacts with RNA at the 2-fold and 3-fold vertices but not at the 5-fold vertices. The absence of density for the RNA at the five-fold vertices can indicate that the RNA and protein capsid do not interact, or that RNA–protein conformations are so dynamic that the density is not observed. The density showed 6 distinct different classes of RNA shapes at the 2- and 3-fold vertices. The different shapes for the RNA at the 2- and 3-fold vertices provide positive evidence for different RNA conformations. Although the absence of density can be interpreted as either multiple conformations or may be the result of limited analysis methods, the distinctly differently-shaped densities for RNA at the 2- and 3-fold vertices likely represent differently structured RNA conformations. The RNA–protein interactions appear to be largely electrostatic interactions, and thus support the hypothesis for a model of virus assembly that invokes electrostatic collapse of the RNA genome and subsequent ordering of protein capsids around the genome. The resulting model of a partially ordered viral genome lies closer to the unstructured end of the spectrum. The genome is disordered but has some places of structure that interact with the protein capsids and help stabilize the virus particle structure and assembly.

On the other hand, the asymmetric reconstruction of MS2 bacteriophage supports a model of virus assembly that nucleates at the RNA interactions with the maturation protein, followed by capsid protein interactions with RNA hairpin packaging signals [4]. When the capsid protein dimer binds an RNA hairpin, a conformation change occurs that facilitates formation of 5-fold or 6-fold vertices by the protein capsids. The asymmetric reconstruction of MS2 virus particles at 3.6 Å reveals 80% of the RNA genome, long-range base pairs, RNA tertiary interactions, and 16 RNA hairpins that can be resolved at the level of individual nucleotides [4]. The asymmetric reconstruction generated 10 different classes of RNA structures that were very similar, with some variation in flexible segments of the RNA. One of the 16 well-defined hairpins binds the maturase protein, and three hairpins form at adjacent 2-fold axes. The 16 RNA hairpins cluster to form a potential nucleation site for virus assembly. Interestingly, the RNA was more densely packed on the side of the particle proximal to the maturation protein, and also provided a structural basis for hypotheses about why the RNA genome is released only when bound to the F1 pili. Although the RNA genome is not in a single conformation, the model lies closer to the highly-structured end of the spectrum and proposes an active role for RNA structure to participate in directing virus assembly.

## 3. Advances in Chemical Probing Sequencing and Analysis Provide New Insights into Viral RNA Ensembles

**Figure 2:**
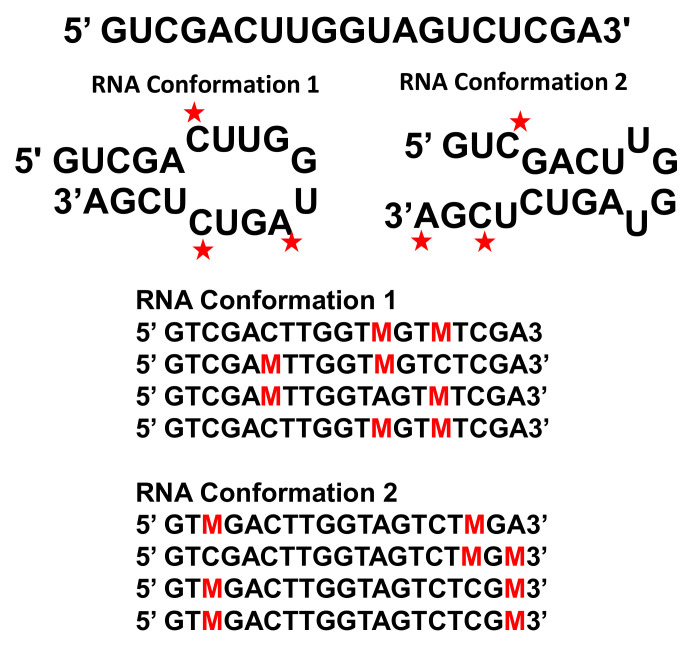
Example of DREEM (Detection of RNA folding Ensembles using Expectation Maximization) analysis of an RNA Hairpin in Two Conformations. The same sequence can fold into two different hairpins. The ensemble average of all the DMS (Dimethyl sulfate) chemical probing hits would show a completely unstructured sequence. Each unpaired nucleotide modified by DMS is indicated by a red star. The DMS modification rate is 2–10% at each nucleotide and varies with local chemical environment. After reverse transcription with TGIRT-III enzyme, the modified nucleotides are read through as a mutation (M). The sequencing reads are grouped according to which mutations occur together. Note that the DMS chemical probing is not 100% efficient and that nucleotides at the ends of helices may also sometimes be weakly modified. This sequence and example are adapted from Figure 1a in reference [6].

Detection of RNA folding Ensembles using Expectation Maximization (DREEM) presents a new approach to studying nucleotide chemical modification with Illumina sequencing [6]. Dimethyl sulfate (DMS) chemically modifies adenines and cytosine bases that are not Watson–Crick paired in the middle of a helix, and this information can be used to constrain RNA folding predictions [25]. DMS-MaPseq [26] reads these methylations using the TGIRT-III enzyme, a reverse transcriptase enzyme that reads through methylations and adds a random mutation at the site of DMS methylation when making a cDNA copy of the RNA template. If more than one DMS methylation occurs on a single strand of RNA, then the TGIRT-III enzyme reads through all of the methylations and does not stop at the methylation as in previous chemical modification strategies. The cDNA can then be analyzed using Illumina sequencing. Previous chemical probing analyses could not distinguish cases in which an RNA adopted more than one conformation, because all of the chemical probing hits were interpreted as an ensemble average and read as a stop in the reverse transcription reaction [27]. If one RNA adopted two conformations, one could not distinguish to which of the two conformations to attribute a chemical modification. With DREEM, each read can include the mutations from chemical modifications for each conformation. DREEM directly clusters experimental data on which nucleotides were methylated prior to modelling the RNA using RNAStructure [28] (Figure 2). For example, if two mutations have a high frequency but never occur on the same read, then this observation supports a model that includes two distinct conformations. Thus, DREEM can identify chemical modifications that occur together in the same RNA conformation and use this information to accurately generate secondary structure predictions for RNA in more than one conformation.

The DREEM clustering approach uses a Bernoulli mixture model that assumes each sequencing reads is a random draw from models that include different subpopulations. The expectation maximization algorithm first assigns the sequencing reads to clusters, then calculates an expected likelihood of observing the data according to the model parameters, and then re-estimates the model parameters to maximize the expected value likelihood. After the iterations of the algorithm converge, the data are normalized and formatted for inclusion as chemical probing constraints in RNAStructure software. The final weight and distribution of each cluster provides an estimate of the relative abundance of each structural conformation. The details of the DREEM algorithms are described in the supporting information of Tomeszko et al. [6], and the authors make the code publicly available at https://codeocean.com/capsule/0380995/tree.

DREEM was applied to the study of HIV RNA in vitro, in virio, and in vivo in HEK293T and CD4+T cells [26]. DREEM was able to assess the equilibrium between two conformations of the Rev Responsive Element (RRE). One conformation has four helical stems coming from a central loop, and another conformation has five helical stems from the central loop. Interestingly, a similar ratio of the two structures in equilibrium occurs in all three environments: 72:28 in vitro, 65:35 in virio, and 73:27 in vivo [6]. The in vitro conditions were 300 mM sodium cacodylate and 6 mM MgCl_2_ at pH 7 and 37 °C after reannealing from 95 °C and equilibrating at 37 °C to generate a thermodynamically-determined conformational ensemble. Surprisingly, these in vitro chemical probing conditions yielded a similar conformational population distribution as the in virio conditions (phosphate buffered solution with 10 mM Tris pH 7 and 3 mM MgCl_2_) and the in vivo cell culture conditions with standard media. The similar ratios of the two RRE conformations supports the hypothesis that the RRE conformational equilibrium is thermodynamically driven and does not depend on specific cellular conditions. RNA folds cotranscriptionally in vivo, and the viral replication process may be kinetically controlled. However, these DREEM results suggest that a locally-folded, thermodynamically-stable conformational ensemble may form during kinetically controlled cotranscriptional in vivo folding.

DREEM has also recently been applied to the study of SARS-CoV-2 RNA in vivo in Vero cells [29]. DREEM is able to identify both conformations of the frameshift element that determines the protein translation of the RNA dependent RNA polymerase (RDRP). The in vivo model for the frameshift element from DREEM differs from previous structures of short in vitro models of the frameshift element [30]. The frameshift element causes translation to shift register by -1 nucleotide, and this shift occurs between open reading frames 1a and 1b. When the frame shift occurs, the frame of a stop codon at the end of ORF1a shifts and the RDRP is then translated. Frameshift elements in both SARS-COV-1 and SARS-CoV-2 are drug targets for small molecule inhibitors [31], and the secondary structure model for the frameshift element has several loop regions that could stabilize one conformation over another and disrupt the viral protein translation regulation. Thus, DREEM can model RNA with more than one conformation, measure functionally important RNA conformation changes in vivo, and identify RNA drug targets in vivo.

## 4. Future Perspectives

Thus, advances in cryoelectron microscopy and chemical probing techniques are providing more insight into the way that a virus folds an RNA genome inside a virus particle. Methods to combine both cryoelectron microscopy and chemical probing data will further refine models for encapsidated viral RNA. Although DREEM uses short 150-nucletoide reads in Illumina sequencing methods, the use of long-read, single molecule sequencing techniques, such as Pac Bio or Oxford nanopore technology, will further enable the identification of chemically accessible nucleotides that are far apart in the sequence but still may change chemical accessibility in a coordinated way, and thus indicate more than one conformation of an RNA molecule. More accurate and detailed models for encapsidated viral RNA will provide a foundation for further testing hypotheses of kinetic or thermodynamic control of virus assembly and the determination of virus assembly mechanisms.
